# Transmission of severe acute respiratory syndrome coronavirus 2 (SARS-CoV-2) to animals: an updated review

**DOI:** 10.1186/s12967-020-02534-2

**Published:** 2020-09-21

**Authors:** Sina Salajegheh Tazerji, Phelipe Magalhães Duarte, Parastoo Rahimi, Fatemeh Shahabinejad, Santosh Dhakal, Yashpal Singh Malik, Awad A. Shehata, Juan Lama, Jörn Klein, Muhammad Safdar, Md. Tanvir Rahman, Krzysztof J. Filipiak, Alfonso J. Rodríguez-Morales, Md. Abdus Sobur, Farrokhreza Kabir, Bita Vazir, Leonard Mboera, Marco Caporale, Md. Saiful Islam, John H. Amuasi, Rasha Gharieb, Paola Roncada, Sahar Musaad, Bruno Tilocca, Mohammad Kazem Koohi, Ali Taghipour, Ahmet Sait, Kannan Subbaram, Alireza Jahandideh, Pejman Mortazavi, Mohammad Amin Abedini, David A. Hokey, Unarose Hogan, Mohamed N. F. Shaheen, Ahmed Elaswad, Mahmoud M. Elhaig, Mohamed Fawzy

**Affiliations:** 1 Young Researchers and Elites Club, Science and Research Branch, Islamic Azad University, Tehran, Iran; 2 Faculty of Biological and Health Sciences, Universidade de Cuiabá (UNIC), Primavera Do Leste, MT Brazil; 3 Faculty of Veterinary Medicine, Science and Research Branch, Islamic Azad University, Tehran, Iran; 4 Kerman University of Medical Sciences, Kerman, Iran; 5 W. Harry Feinstone Department of Molecular Microbiology and Immunology, The Johns Hopkins Bloomberg School of Public Health, Baltimore, MD USA; 6 Division of Biological Standardization, ICAR-Indian Veterinary Research Institute, Izatnagar, Bareilly, Uttar Pradesh India; 7 Research and Development Section, PerNaturam GmbH, 56290 Gödenroth, Germany; 8 Avian and Rabbit Diseases Department, Faculty of Veterinary Medicine, University of Sadat City, Sadat City, Egypt; 9 RetroVirox, Inc., San Diego, CA USA; 10 Faculty of Health and Social Sciences, University of South-Eastern Norway, Kongsberg, Norway; 11 Department of Breeding and Genetics, Cholistan University of Veterinary & Animal Sciences, Bahawalpur, Pakistan; 12 Department of Microbiology and Hygiene, Faculty of Veterinary Science, Bangladesh Agricultural University, Mymensingh, 2202 Bangladesh; 13 Department of Cardiology, Medical University of Warsaw, Warsaw, Poland; 14 Grupo de Investigacion Biomedicina, Faculty of Medicine, Fundacion Universitaria Autonoma de las Americas, Pereira, Risaralda Colombia; 15 Department of Clinical Science, Faculty of Specialized Veterinary Sciences, Science and Research Branch, Islamic Azad University, Tehran, Iran; 16 Department of Physiology, Faculty of Specialized Veterinary Sciences, Science and Research Branch, Islamic Azad University, Tehran, Iran; 17 Emerging and Vector-borne Diseases Program, SACIDS Foundation for One Health, Sokoine University of Agriculture, Morogoro, Tanzania; 18 Istituto Zooprofilattico Sperimentale dell’Abruzzo e del Molise “G. Caporale”, Teramo, Italy; 19 Global Health, and Infectious Diseases Research Group, Kumasi Collaborative Center for Research in Tropical Medicine, Kumasi, Ghana; 20 Department of Zoonoses, Faculty of Veterinary Medicine, Zagazig University, Zagazig, Sharkia Province Egypt; 21 Department of Health Sciences, University “Magna Græcia” of Catanzaro, Catanzaro, Italy; 22 Kanad Hospital, Alain, P.O. Box 1016, Abu Dhabi, UAE; 23 Department of Comparative Biosciences, Faculty of Veterinary Medicine, University of Tehran, Tehran, Iran; 24 Department of Clinical Science, Faculty of Veterinary Medicine, Karaj Branch, Islamic Azad University, Karaj, Iran; 25 Virology Department, Pendik Veterinary Control Institute, Ministry of Food and Forestry, 34890 Pendik-Istanbul, Turkey; 26 Department of Preparatory (Biology), Al-Ghad International Colleges for Applied Medical Sciences, Riyadh, Saudi Arabia; 27 Pathobiology Department, Faculty of Veterinary Medicine, Science and Research Branch, Islamic Azad University, Tehran, Iran; 28 Aeras, Rockville, MD, USA; 29 Infection Prevention and Control, Technical Unit, Americares, Stamford, UK; 30 Environmental Virology Laboratory, Water Pollution Research Department, National Research Division, National Research Center, Dokki, Giza, 12622 Egypt; 31 Department of Animal Wealth Development, Faculty of Veterinary Medicine, Suez Canal University, Ismailia, 41522 Egypt; 32 Department of Animal Medicine (Infectious Diseases), Faculty of Veterinary Medicine, Suez Canal University, Ismailia, 41522 Egypt; 33 Department of Virology, Faculty of Veterinary Medicine, Suez Canal University, Ismailia, 41522 Egypt

**Keywords:** Coronavirus, COVID-19, SARS-CoV-2, Pandemic, Zoonoses, Pet animals, Animals, Epidemiology, One Health

## Abstract

COVID-19 caused by a novel severe acute respiratory syndrome coronavirus 2 (SARS-CoV-2) originated in Wuhan (Hubei province, China) during late 2019. It has spread across the globe affecting nearly 21 million people with a toll of 0.75 million deaths and restricting the movement of most of the world population during the past 6 months. COVID-19 became the leading health, economic, and humanitarian challenge of the twenty-first century. In addition to the considerable COVID-19 cases, hospitalizations, and deaths in humans, several cases of SARS-CoV-2 infections in animal hosts (dog, cat, tiger, lion, and mink) have been reported. Thus, the concern of pet owners is increasing. Moreover, the dynamics of the disease requires further explanation, mainly concerning the transmission of the virus from humans to animals and vice versa. Therefore, this study aimed to gather information about the reported cases of COVID-19 transmission in animals through a literary review of works published in scientific journals and perform genomic and phylogenetic analyses of SARS-CoV-2 isolated from animal hosts. Although many instances of transmission of the SARS-CoV-2 have been reported, caution and further studies are necessary to avoid the occurrence of maltreatment in animals, and to achieve a better understanding of the dynamics of the disease in the environment, humans, and animals. Future research in the animal–human interface can help formulate and implement preventive measures to combat the further transmission of COVID-19.

## Introduction

**Fig. 1:**
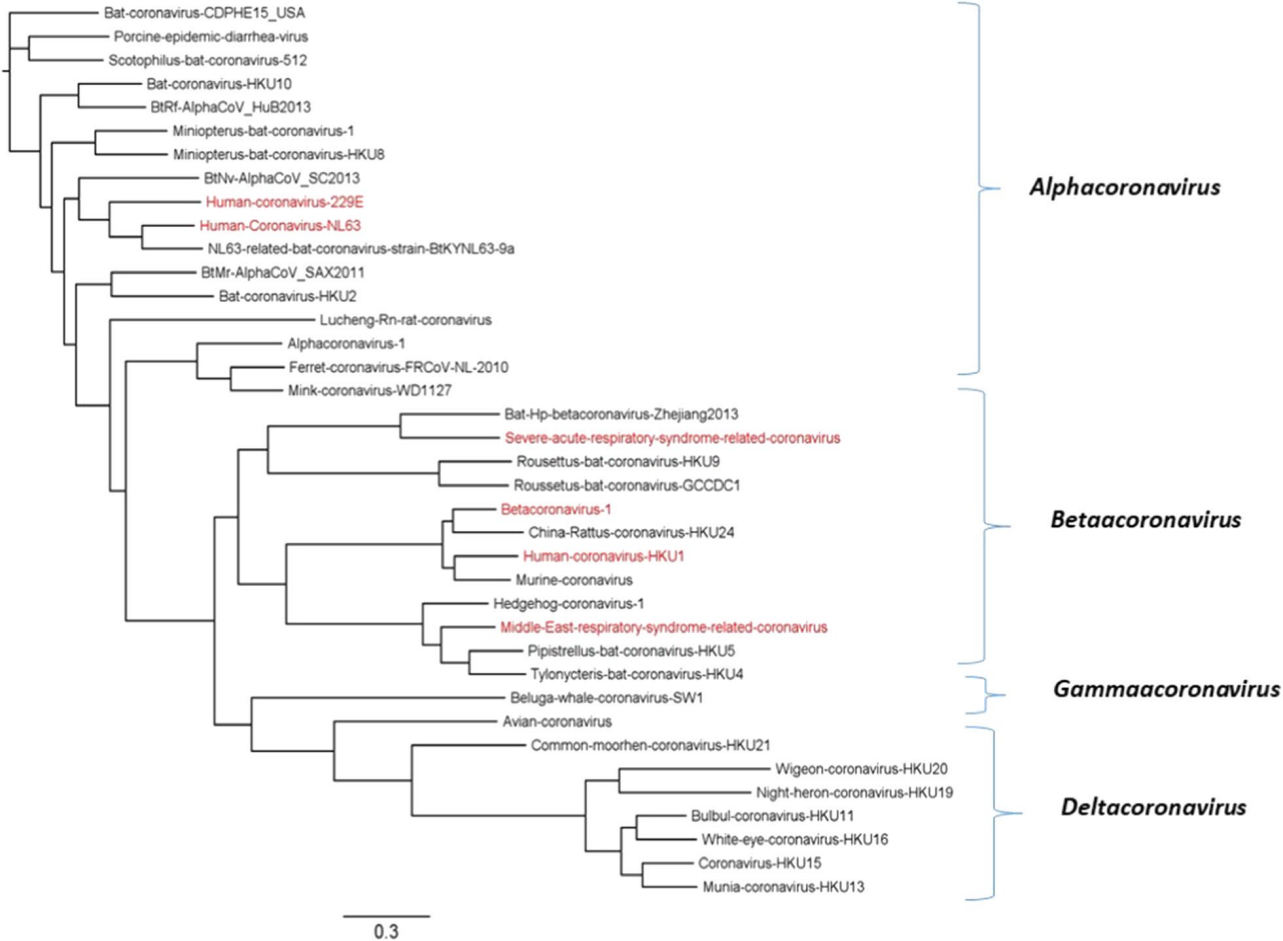
Phylogenetic tree of *Coronaviridae* taxonomy

The outbreak of Coronavirus disease-2019 (COVID-19) leading to pneumonia of unknown origin was linked to the Huanan Seafood Wholesale Market located in the city of Wuhan, Hubei province, China [1, 2]. The pathogen was soon identified as a novel coronavirus named as severe acute respiratory syndrome coronavirus 2 (SARS-CoV-2) and the disease was referred to as Coronavirus disease-2019 (COVID-19) [3]. The SARS-CoV-2 is the third coronavirus that has emerged in the last two decades. Others are the severe acute respiratory syndrome coronavirus (SARS-CoV) and the Middle East respiratory syndrome coronavirus (MERS-CoV), which emerged during 2002 and 2012, respectively [2, 4]. Compared to SARS-CoV and MERS-CoV, SARS-CoV-2 is responsible for the most significant economic loss and the highest number of infections and deaths [5]. Until 13 August 2020 around 21 million COVID-19 cases and over 0.75 million deaths have been reported. SARS-CoV-2 belongs to the order *Nidovirales*, suborder *Coronavirineae*, and family *Coronaviridae*. The family *Coronaviridae* contains two subfamilies named *Lentovirinae* and *Orthocoronavirinae*. The latter is further classified into four genera, namely *Alphacoronavirus* (αCoV), *Betacoronavirus* (βCoV), *Gammacoronavirus* (γCoV), and *Deltacoronavirus* (δCoV), as shown in Fig. 1. The γCoVs and δCoVs cause diseases in birds while αCoVs and βCoVs are mainly found in mammals such as bats, rodents, civets, pigs, horses, cattle and humans [6–9]. SARS-CoV-2 clusters with lineage βCoV together with SARS-CoV and MERS-CoV [8]; both originating from bats [1, 10, 11].

Coronaviruses are enveloped viruses with single-stranded positive-sense RNA (+ss) and genome size between 26 and 32 kb in length [12, 13]. These viruses possess four essential structural proteins: spike glycoprotein (S), matrix protein (M), envelope protein (E), and nucleocapsid protein (N) [13–15, 22]. According to the genomic analysis, SARS-CoV-2 shares a 96.2% identity with the genome of bat CoV; RaTG13, indicating a possible origin of the virus in bats [3, 16]. Several studies suggested that the pangolin could be the potential intermediate host involved in the evolution of the virus because of the unique receptor-binding domain configuration [17–19, 22].

Among the global fear due to the rapid spread of the COVID-19 and absence of specific treatment or vaccine, the first case of human-to-animal transmission was recorded in Hong Kong, where a 17-years-old Pomeranian dog was affected [20]. This case raised concerns about the possibility of SARS-CoV-2 transmission from humans to animals and vice versa, which would represent the difficulties in fighting the virus significantly. However, based on recently published findings, other authors hypothesized that an immunological cross-protection between SARS-CoV-2 and canine respiratory coronavirus (CRCoV) exists due to the high homology between the spike protein epitopes of the two taxonomically-related coronaviruses [21].

The objective of the present study was to gather, present, and discuss information on the reported cases of COVID-19 in animals focusing on the virus transmission cases in pets and perform genomic and phylogenetic analyses of SARS-CoV-2 isolated from animal hosts. Further studies on the dynamics of the disease are essential to adopt suitable control measures to reduce transmission of the virus.

### Possible origin of the new coronavirus

**Fig. 2:**
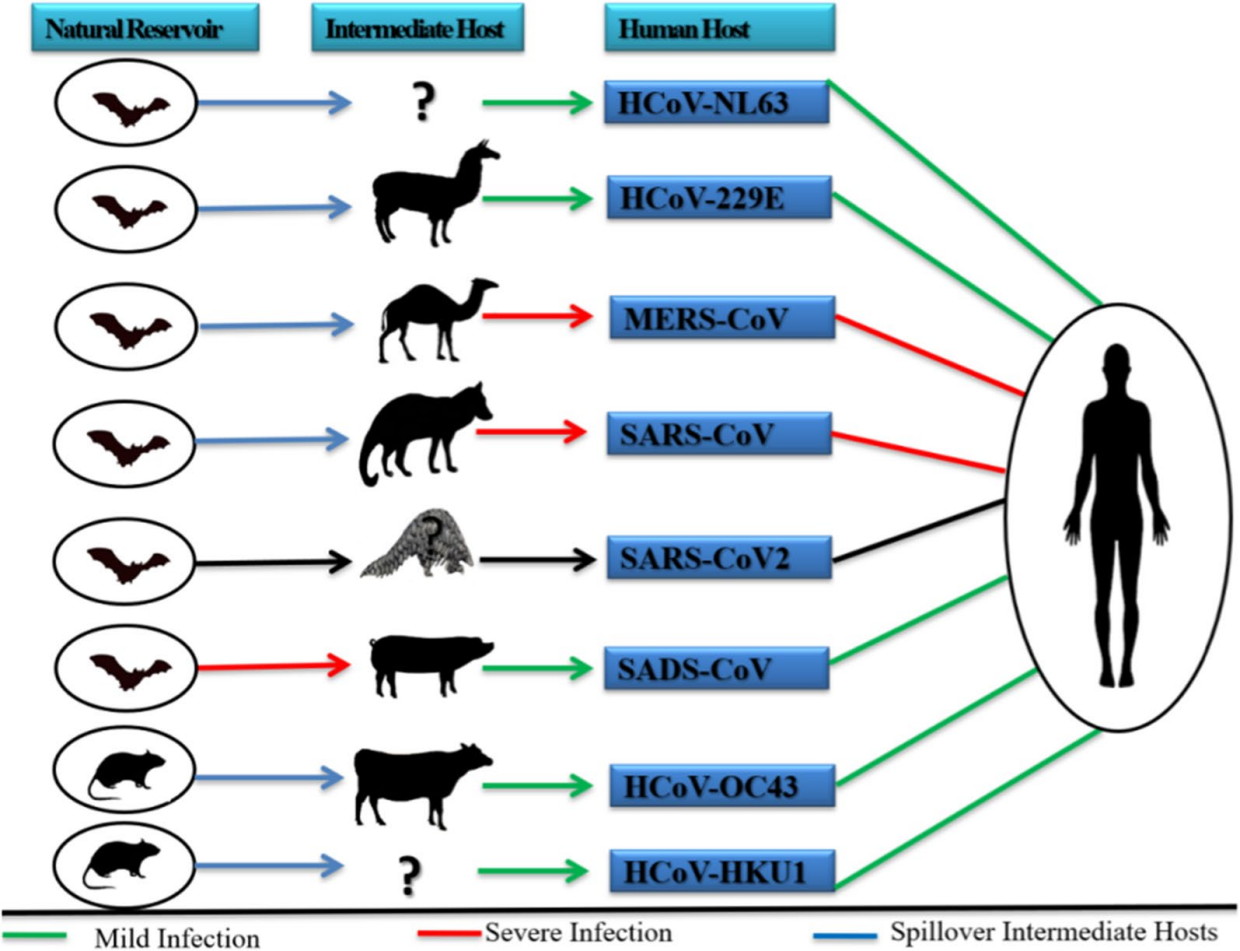
Coronavirus origins and relationship between humans and animals

To discuss the origin of SARS-CoV-2, it is necessary to analyze the source of other coronaviruses such as SARS-CoV and MERS-CoV, as shown in Fig. 2 [22]. SARS-CoV emerged in Guangdong province, Southern China, in November 2002 and was characterized as a contagious respiratory disease of humans [22, 23]. Records showed 8422 registered cases and 916 deaths in humans, spreading to 29 countries during the epidemic with a case fatality rate (CFR) of 9.6% [23]. Studies revealed the presence of several coronaviruses in species of horseshoe bats (genus *Rhinolophus*), which are evolutionarily related to SARS-CoV in their genome organization and sequence [24, 25]. In the further investigations of the initial SARS outbreak of 2002 in Hong Kong, it was recognized that cats (*Felis domesticus*) and ferrets (*Mustela furo*) could be infected with SARS-CoV [26].

In 2012, another coronavirus causing severe acute respiratory syndrome in humans was first reported in Saudi Arabia and was later called the Middle East respiratory syndrome coronavirus (MERS-CoV) [27]. MERS-CoV is βCoV of the C lineage with a genotype very similar to that of bats of the same line, such as BtCoV-HKU4 and BtCoVHKU5 [28].

The transmission of MERS-CoV to dromedaries was identified through the detection of specific antibodies against the virus in these animals [29]. According to Corman et al. [30], MERS-CoV has been circulating in dromedaries for more than 20 years [30]. Therefore, it can be assumed that bats are reservoirs for several coronaviruses, including SARS-CoV, MERS-CoV, and SARS-CoV-2 [25, 31, 32].

According to reports SARS-CoV-2 is closely related to SARS-CoV [33, 34] and the similarity between the genomes was about 80% [3, 16, 35]. Though SARS-CoV-2 has most likely originated from bats, it is not yet clear which animal served as an intermediate host and contributed towards the evolution of the virus before the spillover to humans occurred [22].

In another study, it was demonstrated that SARS-CoV-2 was a chimeric virus between a bat coronavirus and a coronavirus of unknown origin [36]. In a similar research, it was reported a novel bat-derived coronavirus, denoted RmYN02, identified from the metagenomic analysis of samples from 227 bats collected from Yunnan province in China between May and October 2019 [37]. Notably, RmYN02 shares a 93.3% nucleotide identity with SARS-CoV-2 at the scale of the complete genome and 97.2% identity in the ORF1ab gene. It could, therefore, be considered the closest relative of SARS-CoV-2 reported to date. In contrast, RmYN02 showed low sequence identity (61.3%) with SARS-CoV-2 in the receptor-binding domain (RBD), suggesting that RmYN02 might not bind to angiotensin-converting enzyme 2 (ACE2). Critically, and in a similar manner to SARS-CoV-2, RmYN02 was characterized by the insertion of multiple amino acids at the junction site of the S1 and S2 subunits of the spike (S) protein. This provides strong evidence that such insertion events can occur naturally in animal βCoV [37, 39].

It can be seen that tiger SARS-CoV-2/USA/NY-040420 EPI_ISL_420293 shares 99.96% nucleotide identity with human SARS-CoV-2 reference genome at the complete genome-scale, followed by mink SARS-CoV-2/NB04 EPI_ISL_447634 (99.90%), mouse SARS-CoV-2/HRB-26 m EPI_ISL_459910 (99.87%), cat SARS-CoV-2 EPI_ISL_437349 (99.85%), and dog SARS-CoV-2/HKG/20-03695/2020 (99.51%) demonstrating a high level of nucleotide identity between SARS-CoV-2 isolates from domestic animals and humans (Table 1).

**Table 1:** Whole-genome nucleotide identity percent among different representative coronaviruses and human SARS-CoV-2

	1	2	3	4	5	6	7	8	9	10	11	12	13
1-SARS-CoV-2 Reference genome	*100*	99.90	99.51	99.96	99.85	99.87	93.06	95.99	89.90	84.84	87.43	87.69	78.20
2-Mink SARS-CoV-2/NB04 EPI_ISL_447634	99.90	*100*	99.53	99.89	99.78	99.86	93.06	95.99	89.89	84.50	87.37	87.63	78.21
3-Dog SARS-CoV-2/HKG/20-03695/2020	99.51	99.53	*100*	99.54	99.39	99.50	93.06	95.71	89.62	84.68	87.03	87.28	78.55
4-Tiger SARS-CoV-2/USA/NY-040420 EPI_ISL_420293	99.96	99.89	99.54	*100*	99.84	99.83	93.07	96.00	89.87	84.82	87.41	87.66	78.20
5-Cat SARS-CoV-2 EPI_ISL_437349	99.85	99.78	99.39	99.84	*100*	99.72	92.93	95.86	89.76	84.72	87.31	87.56	78.07
6-Mouse SARS-CoV-2/HRB-26 m EPI_ISL_459910	99.87	99.86	99.50	99.83	99.72	*100*	93.14	96.03	89.98	84.92	87.42	87.68	78.26
7-Bat coronavirus RmYN02 EPI ISL 412977	93.06	93.06	93.06	93.07	92.93	93.14	*100*	92.35	87.83	82.64	86.42	86.48	77.72
8-Bat coronavirus RaTG13	95.99	95.99	95.71	96.00	95.86	96.03	92.35	*100*	89.83	84.67	87.36	87.55	78.08
9-Pangolin coronavirus EPI ISL 410721	89.90	89.89	89.62	89.87	89.76	89.98	87.83	89.83	*100*	84.46	86.39	86.44	77.64
10-Pangolin coronavirus PCoV GX-P5L	84.84	84.50	84.68	84.82	84.72	84.92	82.64	84.67	84.46	*100*	82.32	82.48	78.04
11-Bat SARS-like coronavirus CoVZXC21	87.43	87.37	87.03	87.41	87.31	87.42	86.42	87.36	86.39	82.32	*100*	97.24	79.44
12-Bat SARS-like coronavirus CoVZC45	87.69	87.63	87.28	87.66	87.56	87.68	86.48	87.55	86.44	82.48	97.24	*100*	79.42
13-SARS coronavirus civet010	78.20	78.21	78.55	78.20	78.07	78.26	77.72	78.08	77.64	78.04	79.44	79.42	*100*

Also, noteworthy is the shared identity between tiger SARS-CoV-2/USA/NY-040420 EPI_ISL_420293 and mink SARS-CoV-2/NB04 EPI_ISL_447634 (99.89%), cat SARS-CoV-2 EPI_ISL_437349 and tiger SARS-CoV-2 USA NY-040420 EPI_ISL_420293 (99.84%), cat SARS-CoV-2 EPI_ISL_437349 and mink SARS-CoV-2/NB04 EPI_ISL_447634 (99.78), cat SARS-CoV- 2 EPI_ISL_43734 and dog SARS-CoV-2/HKG/20-03695/2020 (99.39%), tiger SARS-CoV-2/USA/NY/040420 EPI_ISL_420293 and dog SARS-CoV-2/HKG/20-03695/2020 (99, 54%), and dog SARS-CoV-2/HKG/20-03695/2020 and mink SARS-CoV-2/NB04 EPI_ISL_447634 (99.53%) (Table 1).

Regarding the percentage of nucleotide identity of the spike protein, dog SARS-CoV-2/HKG/20-03695/2020, tiger SARS-CoV-2 USA NY-040420 EPI_ISL_420293, and cat SARS-CoV-2 EPI_ISL_437349 share 99.97% with human SARS-CoV-2 (Table 2).The amino acid identity of the spike protein between dog SARS-CoV-2/HKG/20-03695/2020, tiger SARS-CoV-2 USA NY-040420 EPI_ISL_42029 and cat SARS-CoV-2 EPI_ISL_437349 with SARS-CoV-2 is 99.92% (Table 3).

**Table 2:** Spike gene nucleotide identity percent among different representative coronaviruses and human SARS-CoV-2

	1	2	3	4	5	6	7	8	9	10	11	12	13
1-SARS-CoV-2 Reference genome	*100*	99.92	99.97	99.97	99.97	99.56	72.16	92.86	83.77	83.23	74.99	75.51	72.54
2-Mink SARS-CoV-2/NB04 EPI_ISL_447634	99.92	*100*	99.95	99.95	99.95	99.48	72.09	92.78	83.74	83.18	74.91	75.43	72.49
3-Dog SARS-CoV-2/HKG/20-03695/2020	99.97	99.95	*100*	100	100	99.53	72.14	92.83	83.74	83.20	74.96	75.48	72.52
4-Tiger SARS-CoV-2/USA/NY-040420 EPI_ISL_420293	99.97	99.95	100	*100*	100	99.53	72.14	92.83	83.74	83.20	74.96	75.48	72.52
5-Cat SARS-CoV-2 EPI_ISL_437349	99.97	99.95	100	100	*100*	99.53	72.14	92.83	83.74	83.20	74.96	75.48	72.52
6-Mouse SARS-CoV-2/HRB-26 m EPI_ISL_459910	99.56	99.48	99.53	99.53	99.53	*100*	72.04	92.49	83.40	83.44	74.78	75.30	72.36
7-Bat coronavirus RmYN02 EPI ISL 412977	72.16	72.09	72.14	72.14	72.14	72.04	*100*	71.50	70.26	69.28	73.01	72.80	69.52
8-Bat coronavirus RaTG13	92.86	92.78	92.83	92.83	92.83	92.49	71.50	*100*	83.25	82.76	75.85	76.01	72.53
9-Pangolin coronavirus EPI ISL 410721	83.77	83.74	83.74	83.74	83.74	83.40	70.26	83.25	*100*	79.69	79.17	79.44	72.19
10-Pangolin coronavirus PCoV GX-P5L	83.23	83.18	83.20	83.20	83.20	83.44	69.28	82.76	79.69	*100*	74.61	75.16	72.32
11-Bat SARS-like coronavirus CoVZXC21	74.99	74.91	74.96	74.96	74.96	74.78	73.01	75.85	79.17	74.61	*100*	96.95	71.83
12-Bat SARS-like coronavirus CoVZC45	75.51	75.43	75.48	75.48	75.48	75.30	72.80	76.01	79.44	75.16	96.95	*100*	71.67
13-SARS coronavirus civet010	72.54	72.49	72.52	72.52	72.52	72.36	69.52	72.53	72.19	72.32	71.83	71.67	*100*

**Table 3:** Spike protein amino acid identity percent among different representative coronaviruses and human SARS-CoV-2

	1	2	3	4	5	6	7	8	9	10	11	12	13
1-SARS-CoV-2 Reference genome	*100*	99.84	99.92	99.92	99.92	99.45	72.54	97.41	90.03	92.38	97.91	80.53	75.76
2-Mink SARS-CoV-2/NB04 EPI_ISL_447634	99.84	*100*	99.92	99.92	99.92	99.29	72.38	97.25	89.87	92.22	79.75	80.38	75.61
3-Dog SARS-CoV-2/HKG/20-03695/2020	99.92	99.92	*100*	100	100	99.37	72.46	97.33	89.95	92.30	79.83	80.46	75.68
4-Tiger SARS-CoV-2/USA/NY-040420 EPI_ISL_420293	99.92	99.92	100	*100*	100	99.37	72.46	97.33	89.95	92.30	79.83	80.46	75.68
5-Cat SARS-CoV-2 EPI_ISL_437349	99.92	99.92	100	100	*100*	99.37	72.46	97.33	89.95	92.30	79.83	80.46	75.68
6-Mouse SARS-CoV-2/HRB-26 m EPI_ISL_459910	99.45	99.29	99.37	99.37	99.37	*100*	72.51	96.94	89.64	92.38	79.75	80.38	75.61
7-Bat coronavirus RmYN02 EPI ISL 412977	72.54	72.38	72.46	72.46	72.46	72.51	*100*	72.76	72.76	72.96	76.66	76.66	71.55
8-Bat coronavirus RaTG13	97.41	97.25	97.33	97.33	97.33	96.94	72.76	*100*	93.14	79.84	80.47	76.31	76.47
9-Pangolin coronavirus EPI ISL 410721	90.03	89.87	89.95	89.95	89.95	89.64	72.76	93.14	*100*	87.55	85.38	85.93	75.33
10-Pangolin coronavirus PCoV GX-P5L	92.38	92.22	92.30	92.30	92.30	92.38	72.96	79.84	87.55	*100*	79.75	80.22	76.59
11-Bat SARS-like coronavirus CoVZXC21	97.91	79.75	79.83	79.83	79.83	79.75	76.66	80.47	85.38	79.75	*100*	98.64	75.00
12-Bat SARS-like coronavirus CoVZC45	80.53	80.38	80.46	80.46	80.46	80.38	76.66	76.31	85.93	80.22	98.64	*100*	75.31
13-SARS coronavirus civet010	75.76	75.61	75.68	75.68	75.68	75.61	71.55	76.47	75.33	76.59	75.00	75.31	*100*

However, the spike protein amino acid and nucleotide identity among tiger SARS-CoV-2/USA/NY/040420 EPI_ISL_42029, dog SARS-CoV-2/HKG/20-03695/2020, and cat SARS-CoV-2 EPI_ISL_437349 was 100% (Tables 2 and 3).

This result seems to be related to animal species that have been infected by SARS-CoV-2 through humans (dog, cat, and tiger, at first). Transmission to humans does not seem so likely, however, given the identity between nucleotides and amino acids of the spike protein, transmission between animals seem possible. Because it has a similar genome to other animal coronaviruses, SARS-CoV-2 may have undergone nucleotide mutation when transmitted to animals, expressing amino acids that increased its pathogenicity in animals, especially those related to spike protein [38, 39].

The RBD of SARS-CoV-2 spike protein, which lies in the S1 domain, is a critical element for determining the susceptibility of the new host species. The studies performed on the interaction between the viral RBD with host cellular receptor (ACE2) revealed snakes, pangolins, and turtles as the potential intermediate hosts [40]. Turtles, along with other animal species, are favored animals in the Huanan Seafood Wholesale Market. However, extended studies are needed to prove their associations scientifically [11].

One of the probable intermediate hosts for SARS-CoV-2 is a pangolin. Pangolin-CoV has 91.02% and 90.55% identity to SARS-CoV-2 and BatCoV RaTG13, respectively [33, 35]. Furthermore, the SARS-CoV-2 spike proteins RBD resembles closely Malayan pangolin CoV (Pangolin-CoV) [40]. These findings suggested that pangolins can be the intermediate host for SARS-CoV-2 transmission. Further research is necessary to confirm the origin and transmission dynamics of SARS-CoV-2.

### Positive aspects of coronaviruses transmission from animals to humans

The possible protective effect of pet-ownership against coronaviruses warrants consideration where coronavirus prevalence is high across the pet population. Canine respiratory coronaviruses often occur among dogs. Ownership of an infected pet can lead to the transmission of viruses from animals to humans. Although possible protection caused by the possession of a pet has not yet been found, the frequent occurrence of coronavirus in canines could help the human immune system develop a better response against SARS-CoV-2 [41].

### Transmission of SARS-CoV-2 from COVID-19 positive persons to animals

At the beginning of the SARS-CoV-2 outbreak, it was thought that pets were not susceptible to the SARS-CoV-2. However, a natural infection of a cat was reported in Belgium with traces of the virus identified in the collected samples by PCR. This cat exhibited respiratory difficulty, vomiting, and diarrhea, which may indicate active replication of the virus inside the animal [39, 41, 42]. However, the animal was not examined by a veterinarian, so further evaluation such as serology was necessary. In another study, Shi et al. showed that cats could be not only naturally infected with SARS-CoV-2 but also that adolescent cats artificially inoculated with the virus presented severe histological lesions and died [18]. However, in further studies, dogs exhibited seroconversion, but the virus could not be isolated. The susceptibility of cats and ferrets to SARS-CoV-2 could be attributed to the ACE2 (SARS-CoV-2 receptors) [3, 43]. These receptors are expressed in type II pneumocytes serous epithelial cells of tracheobronchial submucosal glands in ferrets [44]. Moreover, the SARS-CoV-2 spike-contacting regions of ACE2 are similar in both ferrets and cats, differing by only two amino acids [5].

The previous reports have shown that SARS-CoV can infect ferrets and cats [26], which implies that ferrets and cats may also be susceptible to SARS-CoV-2. This possibility might be associated with cases of SARS-CoV-2 transmission to animals. In addition to probably sharing the same origin, bats have significant similarities among their genotypes [3, 25, 31, 32, 35, 36].

Based on SARS-CoV epidemiology, the COVID-19 pandemic raises the alarm that animals may become infected and become potential transmitters to humans. The fear of possible transmission of animals to humans and the fact that many residents were forced to leave their animals behind due to evacuation and quarantine, thinking that they would return soon, generated a large number of animal abandonment [45].

Authorities in Hunan and Zhejiang provinces of China also announced that they would start killing pets found in public to prevent the transmission of the virus. This concern intensified in late February 2020, when dogs in Hong Kong tested positive for the new coronavirus, being considered the first known case of transmission of COVID-19 from humans to animals later, felines [20, 39, 46, 47].

In a recent study, it was investigated the possible protection conferred by previous exposure of individuals to naturally infected animals with coronaviruses taxonomically related to the circulating SARS-CoV-2 [21, 41, 48–50].

The animals were experimentally infected by SARS-CoV-2 via the intranasal route through the receptor ACE2 [51]. Several studies report the use of ACE2 by the new coronavirus as its receptor for cell entry [3, 43]. Dogs were also inoculated, although seroconversion was observed, no virus could be isolated from the inoculated and uninoculated contacts. Further, ferrets showed equal susceptibility to cats, while pigs, chicken, and ducks were found referent to vulnerability [5].

Until now, studies based on RBD domain analysis ruled out the probability of mice, rats, and rabbit’s involvement in the SARS-CoV-2 cycle [52]. The findings on ferrets, orangutans, and monkeys showed a higher affinity of ACE2 with the RBD domain of SARS-CoV-2 S protein [53]. A codon-usage based analysis pointed to snakes as a probable host, although these findings were contradicted by subsequent studies [54].

Some researchers revealed that cats could naturally be infected with other coronaviruses such as feline coronavirus (FCoV) just as canines can be infected with canine coronavirus (CCoV) [55, 56]. These animals probably get infection once the virus binds to the receptor, ACE2 [51].

Another research group conducted a retrospective serological survey on cats infected with SARS-CoV-2 in Wuhan, China. The authors collected 102 samples after the emergence of COVID-19 and included 39 samples collected before the outbreak. Fifteen (14.7%) of 102 serum samples collected after the outbreak were positive for antibodies against the SARS-CoV-2 receptor-binding domain (RBD) by ELISA test. Among the positive samples, 11 had neutralizing antibodies to SARS-CoV-2 with a titer ranging from 1/20 to 1/1080. Twenty-nine out of the 39 serum samples collected before the outbreak were negative. However, no cat that tested positive on serological tests showed any clinical signs. No serological cross-reactivity was detected between SARS-CoV-2 and type I or II of the feline infectious peritonitis virus (FIPV). As the authors suggested, the cat population studied in Wuhan was infected with SARS-CoV-2 after the beginning of the outbreak [57]. In France, a group of 18 veterinary students investigated the spread of the new coronavirus in 21 pets (9 cats and 12 dogs). Eleven cases showed symptoms compatible with COVID-19, while only two confirmed positive for the new coronavirus. Although three of these cats showed clinical signs of respiratory or gastrointestinal disease, no animal was considered positive by RT-PCR or by the presence of specific antibodies to SARS-CoV-2 [51].

In a separate study, the researchers demonstrated that ferrets, cats, and dogs could be experimentally infected by SARS-CoV-2 via the intranasal route [40]. Several studies reported that the SARS-CoV-2 uses the same receptor, ACE2, to enter the respiratory mucosa [3, 5, 43]. This probably indicates the possibility of transmission of SARS-CoV-2 from humans to animals. In a recent experimental study, it was observed that cats infected with SARS-CoV-2 could transmit the virus to naïve cats that come into contact with them [58]. However, whether they can transmit the virus to humans or else, humans can transmit the virus to pets, or other animals are not yet fully understood.

Also, the first non-domesticated animal case of SARS-CoV-2 transmission was from a big cat, Nadia, and a 4-year-old Malayan tiger infected from the COVID-19 positive workers at the Bronx Zoo, New York, United States. This was the first known animal infection in the US and a tiger anywhere in the world, and was confirmed by the US Department of Agriculture (USDA), the US Centers for Disease Control and Prevention (CDC), National Veterinary Services Laboratories, and the Wildlife Conservation Society (WCS) [54, 59, 60]. Consequently, COVID-19 was detected in four tigers and three lions. Mild infection of respiratory distress was also noted in two pet cats in the USA [61].

A new case was reported in Moskva, Moskovskaya Oblast, Russia, where a 5-year-old cat tested positive for SARS-CoV-2. Samples (throat and nasal swabs) were taken from the suspect cat to detect SARS-CoV-2. The lab tests were performed using RT-PCR in real-time with electrophoretic detection of amplification products. The obtained amplification reaction product was sequenced using selected specific primers flanking a 232 bp N gene fragment (ORF1ab) of the SARS-CoV-2. The tests showed 100% identity of the analyzed fragment of the N gene ORF1ab of the SARS-CoV-2. The animals were quarantined [62].

It was previously shown that SARS-CoV does not infect or cause disease in poultry. Because the COVID-19 virus belongs to the same group as SARS-CoV and uses the same ACE2 host cell receptor, it is highly unlikely that poultry is susceptible to COVID-19. Still, it remains to be scientifically proven [63].

**Fig. 3:**
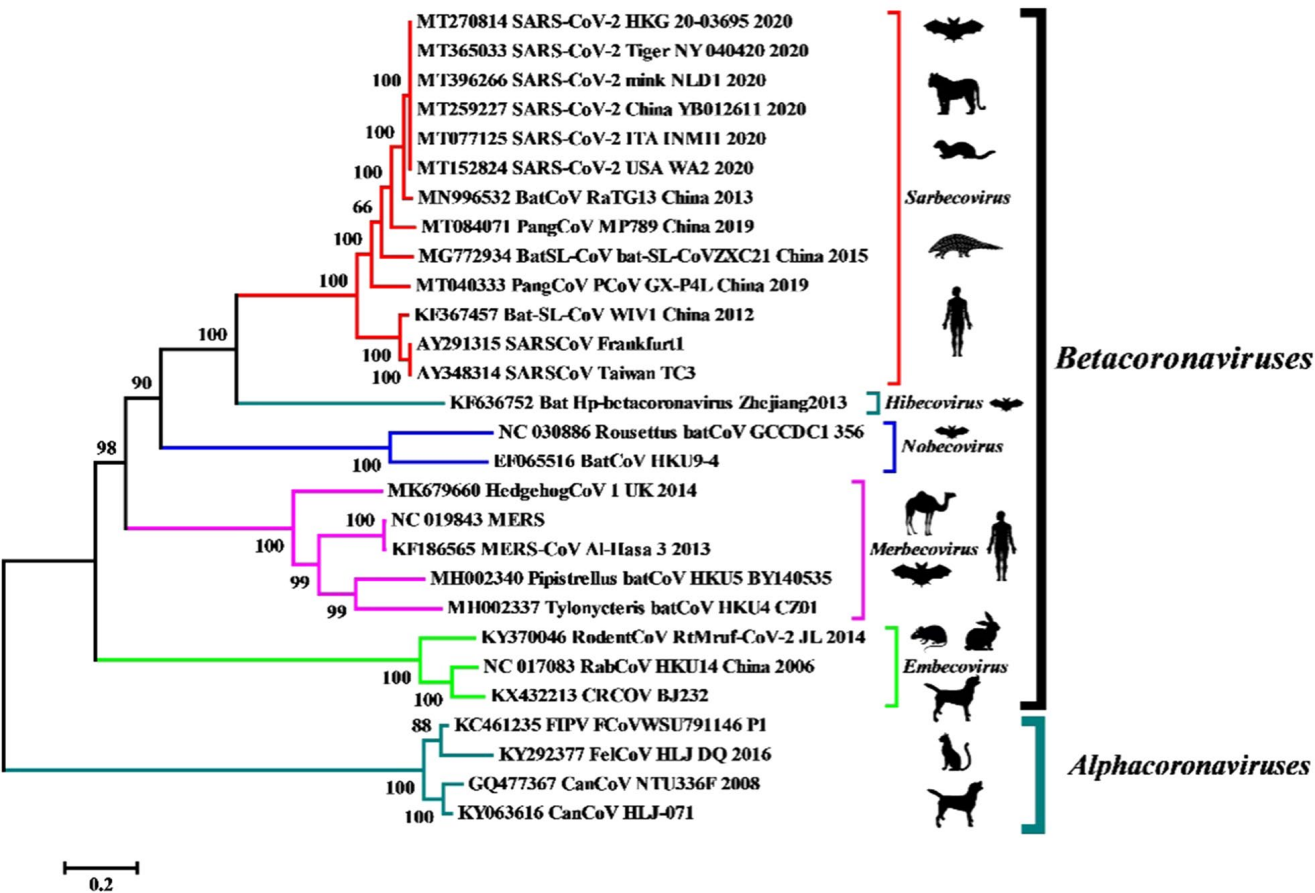
Phylogenetic analysis of human and animal CoVs

For the phylogenetic analysis, complete genome sequences of the viruses falling in the *Betacoronavirus* genus were retrieved from NCBI GenBank. The sequences involve SARS-CoV and SARS-CoV-2 of *Sarbecovirus* subgenus and few of the sequences from different animal species including the dog, mink, rabbit, rat, pangolin, hedgehog, camel, bats, and wild and domestic felines comprising subgenuses *Merbecovirus*, *Embecovirus*, *Hibecovirus* and *Nobecovirus* within the genus *Betacoronavirus* (Fig. 3). The sequence alignment was done with ClustalW in MEGA 6.02 software, and phylogeny was constructed using the GTR + G + I substitution model applying the maximum likelihood method. In the analysis, canine and feline CoVs of Alphacoronavirus were taken as the outgroup for constructing a phylogenetic tree of Betacoronavirus. All the CoVs of genus *Betacoronavirus* clustered in their respective subgenus clades. Notably, the mink and tiger virus isolates showed 99.6–99.9% homology with human SARS-CoV-2 isolates from different parts of the world. Contrarily, the canine Betacoronavirus (CRCoV) from subgenus *Embecovirus* showed 45.8–46.2% similarity with SARS-CoV-2. Furthermore, pangolin CoV, SARS-like bat CoVs, and bat CoVs (RaTG13 strain) showed homology between 86.6 and 96.3% with SARS-CoV-2. The camel isolate of MERS-CoV showed a 51% similarity with SARS-CoV-2 at the nucleotide level (Fig. 3). Our analysis demonstrated a high divergence between animal origin CoVs and SARS-CoV-2 except for a few strains which were isolated from mink and tiger as were the cases of COVID-19 infected persons.

### Auspicious mode of transmission of SARS-CoV-2 from humans to animals

Though the exact ways of transmission of SARS-CoV-2 from infected humans to animals are vaguely understood, the possible and promising transmission may occur through touching their noses or mouth by infected hands defiled with respiratory droplets or saliva [64]. At the time of sneezing, coughing, or even talking, infected humans can disseminate respiratory droplets, which have a pivotal role in the transmission of the virus to animals [65]. However, the transmission of the virus from affected people can be facilitated by some favorable risk factors, e.g., kissing, petting, licking, or hugging pet animals [66].

### Case of animal-to-human transmission of coronavirus at a mink farm in the Netherlands

According to the government of the Netherlands, through a letter issued by the Ministry of Agriculture, Nature and Food Quality, it is possible that an employee who worked on a mink farm infected with SARS-CoV-2 contracted the virus, having already been recovered from the disease. Also, research shows that minks can be asymptomatic and that cats play an important role in the potential transfer of viruses between investigated farms [67].

## Conclusions

There are multiple studies on the origin of coronaviruses and their zoonotic potential. Some coronaviruses that infect animals can sometimes be spread to humans and then spread between people as happened in the case of MERS and SARS. This is also what happened with the virus that caused the current outbreak of COVID-19. However, the exact origins of this virus are still unclear. The scientific community has put significant effort into identifying the source of SARS-CoV-2, and to date, genetic evidence suggests that it was likely acquired from bats. The first infections were linked to a live animal market in Wuhan, suggesting a zoonotic origin. The virus is now spreading from person to person and caused one of the most significant pandemics of the recent era. Infection with SARS-CoV-2 has been reported in several animals through serological and molecular analyses and experimental inoculations. However, it is not proved that animals can transmit SARS-CoV-2 to humans and to what extent humans can transmit this virus to animal species. Veterinary public health officials and designated public health officials should work to determine whether animals should be tested for SARS-CoV-2 when the animals are in the same environment as infected owners using a One Health approach. Available information suggests that the risk of SARS-CoV-2 spread from animals to humans is low. Even though human-to-animal transmission or vice versa is possible, caution and effective communication with the pet owners are necessary to prevent the abandonment and death of animals indiscriminately. Although a case of SARS-CoV-2 transmission from animals to humans has already been reported, this is only a report, so further research is needed before declaring that animals can transmit the new coronavirus to humans. Geographic information system (GIS) based on computer and information technology would be useful in analyzing the disease pattern. The collaborative global effort, such as “One-Human–Environmental–Animal Health,” is required to reduce the global risk of zoonotic diseases. Further studies are essential to understand better the origin of the virus and transmission dynamics, which will help educate people and avoid unnecessary discrimination to animals. Preventive measures that have proven to be effective should be encouraged, such as correct hygiene, social distance, and, if necessary, social isolation or quarantine. Good practices such as the use of footbaths, whirlpools, disinfection of urban areas, and sanitary barriers can be considered.

## Data Availability

All data analyzed or generated during this study are included in this publication and its additional files.

## Abbreviations

COVID-19: Coronavirus disease-2019
SARS-CoV-2: Severe acute respiratory syndrome coronavirus 2
MERS-CoV: Middle East respiratory syndrome coronavirus
αCoV: *Alphacoronavirus*
βCoV: *Betacoronavirus*
γCoV: *Gammacoronavirus*
δCoV: *Deltacoronavirus*
CRCoV: Canine respiratory coronavirus
Kb: Kilobase
CFR: Case fatality rate
RBD: Receptor-binding domain
ACE2: Angiotensin-converting enzyme 2
PCR: Polymerase chain reaction
ELISA: Enzyme linked immunosorbent assay
FIPV: Feline infectious peritonitis virus
RT-PCR: Real-time polymerase chain reaction
USDA: US Department of Agriculture
CDC: US Centers for Disease Control and Prevention
WCS: National Veterinary Services Laboratories and the Wildlife Conservation Society
IBV: Infectious bronchitis vírus
NCBI: National Center for Biotechnology Information
GIS: Geographic information system
